# A Second Actin-Like MamK Protein in *Magnetospirillum magneticum* AMB-1 Encoded Outside the Genomic Magnetosome Island

**DOI:** 10.1371/journal.pone.0009151

**Published:** 2010-02-10

**Authors:** Jean-Baptiste Rioux, Nadège Philippe, Sandrine Pereira, David Pignol, Long-Fei Wu, Nicolas Ginet

**Affiliations:** 1 Laboratoire de Bioénergétique Cellulaire – Institut de Biologie Environnementale et Biotechnologie, Commissariat à l'Energie Atomique, Saint-Paul-lez-Durance, France; 2 UMR 6191 – Biologie Végétale et Microbiologie Environnementale, Centre National de la Recherche Scientifique, Saint-Paul-lez-Durance, France; 3 Aix-Marseille Université, Saint-Paul-lez-Durance, France; 4 UPR 9043 – Laboratoire de Chimie Bactérienne, Centre National de la Recherche Scientifique, Marseille, France; 5 Laboratoire de Radiotoxicologie et d'Ecotoxicologie, Institut de Radioprotection et de Sûreté Nucléaire, Saint-Paul-lez-Durance, France; Cairo University, Egypt

## Abstract

Magnetotactic bacteria are able to swim navigating along geomagnetic field lines. They synthesize ferromagnetic nanocrystals that are embedded in cytoplasmic membrane invaginations forming magnetosomes. Regularly aligned in the cytoplasm along cytoskeleton filaments, the magnetosome chain effectively forms a compass needle bestowing on bacteria their magnetotactic behaviour. A large genomic island, conserved among magnetotactic bacteria, contains the genes potentially involved in magnetosome formation. One of the genes, *mamK* has been described as encoding a prokaryotic actin-like protein which when it polymerizes forms in the cytoplasm filamentous structures that provide the scaffold for magnetosome alignment. Here, we have identified a series of genes highly similar to the *mam* genes in the genome of *Magnetospirillum magneticum* AMB-1. The newly annotated genes are clustered in a genomic islet distinct and distant from the known magnetosome genomic island and most probably acquired by lateral gene transfer rather than duplication. We focused on a *mamK-like* gene whose product shares 54.5% identity with the actin-like MamK. Filament bundles of polymerized MamK-like protein were observed in vitro with electron microscopy and in vivo in *E. coli* cells expressing MamK-like-Venus fusions by fluorescence microscopy. In addition, we demonstrate that *mamK-like* is transcribed in AMB-1 wild-type and Δ*mamK* mutant cells and that the actin-like filamentous structures observed in the Δ*mamK* strain are probably MamK-like polymers. Thus MamK-like is a new member of the prokaryotic actin-like family. This is the first evidence of a functional *mam* gene encoded outside the magnetosome genomic island.

## Introduction

Magnetotactic bacteria (MTB) are a group of taxonomically, physiologically, and morphologically diverse prokaryotes with the ability to align along geomagnetic field lines [1]–[3]. Intracellular alignments of specialized organelles called magnetosomes are responsible for this behaviour. MTB are usually found in oxic-anoxic transition zones, interfaces between oxygen-rich and oxygen-starved biotopes in fresh and marine waters and aquatic sediments. Many MTB are only able to survive in environments where the oxygen concentration is very low and some can only exist in completely anaerobic conditions [4], [5]. The evolutionary advantage of possessing magnetosomes is possibly linked to the ability to efficiently navigate within zones of such sharp chemical gradients by simplifying a three-dimensional search for conditions of optimal oxygen concentration to a single dimension, north-south. Magnetosomes are composed of single-domain magnetic nanocrystals of magnetite or greigite (35 nm to 120 nm long) embedded in biological membranes. Magnetosomes are regularly aligned inside the cytoplasm and the sum of their respective magnetic moments defines a true compass needle. In the *Magnetospirilla*, the ‘compass needle’ is set parallel to the cell's direction of movement, allowing passive alignment of the cell along geomagnetic field lines [5]. When MTB cells are disrupted, magnetosomes can be readily purified as closed vesicles encircling a single crystal by using magnets. The separation of functional membranes by magnetism elicits great interest in bio- and nanotechnology [6], [7].

The genomes of *Magnetospirillum (M.) magneticum* AMB-1 [8], *M. magnetotacticum* MS-1, *M. gryphiswaldense* MSR-1, *Magnetococcus sp.* MC-1 [9] and *Desulfovibrio magneticus sp.* RS-1 [10] have all been completely sequenced leading to the identification of a large genomic island containing many of the genes potentially involved in magnetosome formation [11]. This genetic element, termed the magnetosome island (MAI), is approximately 100 kb long and is unstable and subject to frequent rearrangement. When the MAI is lost or partially deleted, the bacteria are no longer able to synthesize magnetosomes. Numerous transposable elements, direct repeats and tRNA genes found in MAIs and the lower GC content suggest that horizontal gene transfer is responsible for the spread of magnetotaxis among microorganisms.

Several comparative genomic studies have led to the identification of a minimal set of magnetotaxis-specific genes, shared by all MTB regardless of their phylogeny [12]–[14]. In MTB belonging to the alpha proteobacteria, these 17 genes are *mamH, E, K, M, O, P, A, Q, B, S, T, C, D, Z, X* and *mms6* and *mmsF* (gene names based on *M. gryphiswaldense* gene nomenclature). The degree of similarity between orthologous genes can be very high even for distantly related species, e.g. *mamK* in AMB-1 and *mamK-I* in MV-1 share 50.5% identity. However the genetic organization within the MAIs differs and generally seems to be genus-specific. Within the most studied genus *Magnetospirillum*, genetic differences have been noted such as a partial duplication of the *mamAB* operon in *M. magneticum* AMB-1 and *M. magnetotacticum* MS-1 that is not found in *M. gryphiswaldense* MSR-1. Generally speaking, these duplications seem to be a hallmark of the instability of the MAI, regardless of the species. The consensus is that all the MTB-specific genes cited above are located within the MAI of MTB.

Although more genetic determinants of magnetosome biosynthesis have been identified in the past decade, the molecular mechanisms involved are still poorly understood. Among the MTB-specific genes above cited, we have functional evidences about MamD, involved in the regulation of the size of the crystals [15] and MamA, required for the activation of the magnetosomes [16] and molecular information for only 2 of them, *mms6* and *mamK*. Mms6, a small acidic protein tightly bound to magnetite particles, is involved in magnetite nucleation and controlling crystal shape and size, as demonstrated in vitro [17], [18]. MamK belongs to the family of actin-like proteins [19], [20]. Although phylogenetically distant from eukaryotic actins, bacterial actin-like proteins share structural and functional homologies with them. They have a three-dimensional fold characteristic of actin due to conserved sequence motifs. They also have a nucleotide-binding site in common. Bacterial actin-like proteins belonging to the MreB and ParM families have been studied in most detail [21], [22]. MamK is closely related to the MreB family: it has a nucleotide-binding site and polymerizes into filaments that assemble into bundles extending from one pole of the cell to the other. Magnetosome vesicles are aligned along this scaffold probably via MamK interacting with MamJ [23]. The individual filaments have been visualized in vitro by TEM [24] and the bundles in vivo in *M. magneticum* AMB-1 by electron cryotomography [19] and immuno-gold labelling [20]. Pradel et al. [20] found that MamK nucleates at multiple sites and assembles into mosaic bundles of filaments. The assembly of MamK bundles is highly dynamic and kinetically asymmetrical. Possible functions of MamK filaments in magnetotaxis could be in anchoring magnetosomes or in magnetism perception. MamJ is thought to be involved in the attachment of the magnetosome to MamK filaments: deletion of *mamJ* in *M. gryphiswaldense* MSR-1 causes magnetosomes to aggregate in the cytoplasm, so the alignment characteristic of the wild-type strain is lost [25].

Although widely accepted the MamK-MamJ functional model [26]–[28] may need revising as *mamK* is present but *mamJ* is absent from the recently sequenced *Magnetococcus sp.* MC-1 genome [9]. This prompted us to reanalyze available genomic sequences of *Magnetospirilla*. Intriguingly, we identified a magnetotaxis genomic islet containing seven putative magnetotaxis genes including a *mamK* homologue. We found that the *mamK-like* gene is expressed in *M*. *magneticum* AMB-1 and the recombinant MamK-like protein is able to polymerize in vitro into long filaments assembled into bundles. Its expression alone is sufficient for the assembly of MamK-like bundles in *E. coli* cells. In *M. magneticum* AMB-1 Δ*mamK*, a strain devoid of the *mamK* gene, we observed thin linear structures spanning the cytoplasm that we consider to be composed of MamK-like filaments. This is the first characterization of magnetotaxis genes whose coding sequence is located outside the MAI.

## Materials and Methods

All chemicals were purchased from Sigma-Aldrich unless specified otherwise.

### Bioinformatics

For genomic and proteomic analysis, genomes of *M. magneticum* AMB-1, *Magnetococcus sp.* MC-1 and *M. magnetotacticum* MS-1 were imported into the microbial genome expert annotation system MaGe provided by Genoscope (https://www.genoscope.cns.fr). Other genetic data originating from *M. gryphiswaldense* MSR-1, *Desulfovibrio magneticus sp.* RS-1, marine magnetotactic vibrio strain MV-1, uncultured bacteria 0904b6_Fos001 and mtbm116/Fos002 were used with online data mining tools provided by NCBI (http://www.ncbi.nlm.nih.gov). Genomic data accession numbers and abbreviations used thereafter are summarized in Table 1.

**Table 1:** Genomic data used in this study.

Organism	Abbrev.	Database	Accession number
*M. magneticum* AMB-1	AMB-1	NCBI	NC_007626.1
*M. magnetotacticum* MS-1	MS-1	NCBI	NZ_AAAP00000000.1
*Magnetococcus sp.* MC-1	MC-1	NCBI	NC_008576.1
*M. gryphiswaldense* MSR-1	MSR-1	Genbank	CU459003.1
Marine magnetotactic vibrio MV-1	MV-1	Genbank	FP102531.1
*Desulfovibrio magneticus sp.* RS-1	RS-1	NCBI	NC_012795.1
Uncultured bacterium 0904b6_Fos001	Fos001	Genbank	FP312973.1
Mtbm116/Fos002	Fos002	Genbank	FP312985.1

For simplicity, throughout the paper we used the abbreviations listed in the second column when referring to these organisms.

### Phylogenetic Analysis

Codon usage patterns were analyzed with the General Codon Usage Analysis (GCUA) software [29]. Briefly, a codon usage table is generated and converted into a relative synonymous codon usage value (RSCU), which expresses the codon usage bias for a given residue. A distance matrix is then computed which groups DNA sequences on the basis of the similarity of RSCU values. Using the PHYLIP package [30], a hierarchical tree-like representation of these data can be generated using the Fitch-Margoliash distance-based optimization method. The latter generates *N* (we selected *N* = 100) distance matrixes with a randomized input order of elements and computes the best tree. Protein alignments were computed with ClustalW [31].

### Structural Modelling

To generate 3-D protein models from primary structures we used 3D-JIGSAW, a fully automated protein structure homology-modelling program [32]–[34], scanning the Protein Data Bank for templates [35].

### *Magnetospirillum magneticum* AMB-1 Culture

Wild-type (WT) AMB-1 and the Δ*mamK* mutant strain (kindly provided by Dr. Komeili) were grown in Komeili's medium [16] supplemented with 0.2 g/l soy bean peptone and 0.1 g/l yeast extract. Static cultures were grown in hermetically sealed Schott bottles containing 80 ml of growth medium with a 30 ml headspace; cultures were flushed with a nitrogen/air mixture at 2% O_2_ for 10 min after inoculation and grown at 28°C.

### RNA Extraction and RT-PCR

Cells harvested during the exponential phase were resuspended in 1 volume of growth medium, and 2 volumes of RNAprotect™ Bacteria Reagent (Qiagen) was then added to stabilize the RNA. RNA extraction was performed using the Qiagen RNeasy Minikit. DNA was removed in two steps, on-column using Qiagen RNase-free DNase and after elution using RNase-free DNase (New England Biolabs). RNA was protected with 5 mM EDTA prior to DNase inactivation. cDNA was generated with the Takara RNA PCR Kit (AMV) with 500 ng of RNA as template and random 9-mers. FlexiGoTaq (Promega) was used for PCR amplification and 50 ng of genomic DNA and 100 ng of RNA were used as templates for positive and negative controls respectively. Primers used are summarized in Table 2 (sets 1, 2 and 3 for *mamE*, *mamK* and *mamK*-*like* amplification respectively). Primers were shown not to cross-hybridize to genomic DNA using routine PCR procedures.

**Table 2:** Primer sets used for PCR amplifications.

Set	Forward (5′→3′)	Reverse (5′→3′)
***1***	CGGGGTGCAATCCGTGC	CCAGGGGATCGGGCATG
***2***	GAACGGAGTGACAAAAAT	TCCCGCATATCGAACTCT
***3***	CAGCTAGATTCGGGGACA	GCCAGTAGTGGGCTTATC
***4***	CACCATGAGCGAAGGAGAAGGGCAG	TTACGAGCCCGACACGTCTCC
***5***	CACCATGATGATTGTGAACGATAA	AAGCTGCCCCCAAAAGTGAG
***6***	CGGAATTCACCATGAGTGAAGGTGAAGGCCA	TCCTCGCCCTTGCTCACCATCGAGCCGGAGACGTCTCCAA
***7***	CAGGAGGAATTCATATGATTGTGAACGATAACCA	TCCTCGCCCTTGCTCACCATAAGCTGCCCCCAAAAGTGAG
***8***	TTGGAGACGTCTCCGGCTCGATGGTGAGCAAGGGCGAGG	GCCATTCTAGATTACTTGTACAGCTCGTCCA
***9***	CTCACTTTTGGGGGCAGCTTATGGTGAGCAAGGGCGAGGA	GCCATTCTAGATTACTTGTACAGCTCGTCCA

Each set comprises a forward (left column) and a reverse (right column) primer.

### Protein Purification

Recombinant six-histidine-tagged MamK and MamK-like were produced in *E. coli* BL21 Star™ (DE3) (Invitrogen). *mamK* and *mamK-like* genes were amplified by PCR from AMB-1 genomic DNA using primer sets 4 and 5 respectively (Table 2), and cloned into expression vectors pet100D/TOPO for *mamK* and pet101D/TOPO (Invitrogen) for *mamK-like*. Plasmids were checked by sequencing. MamK-like sequence including the N-terminus histidine-tag was deposited in Genbank database under the accession number **GQ457518**. *E. coli* were transformed with the plasmids and grown in 3 l Terrific Broth medium. Protein expression was induced with 0.25 mM IPTG (final concentration) in the exponential growth phase (OD_600nm_  = 0.6). After overnight culture at 16°C, cells were harvested and disrupted with a French Press cell system, in a lysis buffer (pH 8) containing 100 mM Tris-HCl, 150 mM NaCl, 14 mM MgCl_2_ and 1 mM ATP. DNase and a protease inhibitor cocktail (Sigma-Aldrich) were added. After centrifugation (100 000 g, 1 h), the supernatant was loaded onto a 1 ml His-trap column (GE healthcare). Column washes and protein elution were performed using lysis buffer containing 75 mM and 250 mM imidazole respectively. Protein content was determined by the Bradford assay (CooAssay kit, Interchim).

### Western Blot Analysis

Protein purity was checked by SDS-PAGE (30 ng of each purified protein loaded on 10% polyacrylamide gels) and Western blot. Anti-polyhistidine peroxidase conjugate antibody (Sigma Aldrich) was used at 1∶10000 dilution. Anti-MamK (kindly provided by Prof. Fukumori) was used at 1∶1000 dilution. The secondary antibody anti-rabbit peroxidase conjugate (Sigma-Aldrich) was diluted 1∶5000 in Tris-Buffered Saline.

### *In Vitro* Polymerization of MamK-Like and MamK

Purified MamK and MamK-like proteins were assayed for in vitro polymerization with a protocol adapted from Taoka's [24]. Proteins were desalted on a PD-10 column (GE Healthcare) in 20 mM MES (pH 6), 20 mM NaCl. Aliquots of purified protein (6 µM) were mixed with salt and buffer, giving the final concentrations: 20 mM Tris (pH 7), 30 mM KCl, 75 mM NaCl, 14 mM MgCl_2_. Mixes were incubated for 15, 30 or 60 min at 25°C, then 10 µl of the mix was spotted on formvar/carbon-coated grids, and after 2 min the sample was washed and negatively stained with 1% (w/v) uranyl acetate for 1 min. Grids were observed using a Zeiss EM9 transmission electronic microscope at 80 kV.

### *In Vivo* Polymerization Kinetics of MamK-Like and MamK Fused to Venus

The *E. coli* TG1 strain was used. The Phusion High Fidelity DNA Polymerase (Finnzymes) was used for all PCR reactions. *MamK* and *MamK-like* genes were fused to *venus* encoding the fluorescent protein Venus derived from YFP [36] in a two-step PCR method. First, *mamK* and *mamK-like* were amplified by PCR (primer sets 6 and 7 respectively, see Table 2) yielding fragments #1, then *venus* was amplified (fragments #2) with primer sets 8 and 9 (for *mamK-* and *mamK-like-venus* fusions respectively). Primers sets 6/8 and 7/9 include overlapping sequences to allow subsequent hybridization of fragments #1 and #2. Second, for each gene PCR amplification was performed with equal amounts of purified fragments #1 and #2 as templates and with no additional primers, yielding the fusion genes *mamK-venus* and *mamK-like-venus*. These DNA fragments were digested with *EcoRI* and *XbaI* and cloned into pBAD24, under the control of the *pBAD* promoter. The resulting plasmids were checked by PCR, digestion, and sequencing. TG1 clones carrying the plasmids pMamK-Venus or pMamK-like-Venus were grown in Luria-Bertani medium at 30°C starting from overnight cultures diluted 1∶100. The production of the corresponding fusion proteins was induced by addition of 0.1 or 0.2% (w/v) arabinose during the exponential growth phase. Samples were taken at regular intervals (between 30 min and 18 hours after induction) and fixed in 2% paraformaldehyde in PBS (17.5 mM NaCl, 0.38 mM KCl, 1 mM Na_2_HPO_4_, 0.19 mM KH_2_PO_4_) for 5 min at room temperature then washed twice in PBS. Live or fixed cells were observed with the epifluorescence microscope Axiovert 200 M (Zeiss, Göttingen, Germany) connected to an ORCA ER camera (Hamamatsu, Tokyo, Japan). Excitation wavelength was 515 nm and fluorescence emission was monitored at 528 nm. The exposure time was determined according to the fluorescence intensity going from 1000 ms at the beginning to 50 ms at the end of the time-course. Fluorescent images were deconvoluted using the Imaris software package (Bitplane, Zürich, Switzerland) and Huygens Essential software (Scientific Volume Imaging, Hilversum, The Netherlands).

### Immunofluorescence

The immunofluorescence protocol used has been described [24]. Briefly, AMB-1 WT or *ΔmamK* mutant cells were harvested (50 ml culture, OD_600nm_  = 0.1), concentrated and fixed on microscope slides in 4% (w/v) paraformaldehyde, 2% (w/v) sucrose in Phophate-Buffered Saline (PBS) (Invitrogen) for 15 min at room temperature, kept for 45 min at 4°C and permeabilized in 20 mM HEPES (pH 7.4) 50 mM NaCl, 3 mM MgCl_2_, 300 mM sucrose, 0.5% (v/v) Triton X-100 for 10 min. Slides were washed in PBS prior to immunostaining. Primary antibody incubations were done overnight at 4°C in PBS supplemented with 2% (w/v) bovine serum albumin fraction V and followed by a PBS wash. The anti-MamK antiserum was used at a 1∶100 dilution. Mouse anti-rabbit IgG coupled to fluorescein (FITC) diluted 1∶100 was incubated on slides at 37°C for 20 min. Bacterial membranes were stained with FM® 4-64 FX, a fixable membrane stain (Molecular Probes). Slides were mounted in 4′,6-diamidino-2-phenylindole-stained Vectashield (Abcys) and examined with a Nikon Eclipse 6000 microscope. Cells were then observed by confocal microscopy with a Leica TCS SP5 microscope using a planAPOchroma oil immersion 100x objective. The 488 nm Ar/Kr and 633 nm He/Ne emission bands were used for FITC and FM® 4-64 FX excitation respectively. Fluorescence was captured around the maximum emission wavelengths, i.e. 518 nm and 744 nm for FITC and FM® 4-64 FX respectively. Three-dimensional stacks of 2048 x 2048-pixel images were acquired using a 0.16-µm step after 4x frame average and 2x line accumulation with a 2.4x or 4.74x zoom in order to get the best resolution and the best signal-to-noise ratio.

### TEM to Visualize Magnetosome Alignment

AMB-1 cells were grown in EMSGM medium [37] for 48 h and concentrated to an OD_600nm_ of 1. Aliquots of 10 µl were spotted on formvar-carbon coated grids and after 2 min were negatively stained with 1% (w/v) uranyl acetate for 1 min if needed. Grids were observed using a Zeiss EM9 transmission electronic microscope at 80 kV.

## Results

### A Genomic Islet Outside the MAI Encodes Putative *mam* Genes

Redundancy in the functions of several magnetotaxis-specific genes in MAIs of different magnetotactic bacteria have been shown, suggestive of frequent genetic rearrangement (for a convenient comparison of available genome sequences, see [13]). We focused on the genome of *M. magneticum* AMB-1 and sought additional homologues of the *mam* genes in the entire genome. Using the MaGe interface for expert annotation of microbial genomes, we identified a new locus of approx. 22 kb outside the MAI, containing seven ORFs similar to genes from the *mamAB* and *mamCD* operons. We will hereafter refer to these newly identified magnetotaxis-related genes as *mam-like* genes. Similarities between the proteins encoded by these putative genes and those present in the *mamAB* and *mamCD* operons clustered in the MAI of AMB-1 are very high (75.6% sequence identity between MamL and MamL-like, 54.5% between MamK and MamK-like (*NEW_GENE*), and 51.9% between MamE and MamE-like, see Table 3). MamJ-like is a notable exception having less than 20% identity with MamJ. Despite this, we are confident that the gene is assigned correctly: two short amino-acid motifs shared by all MamJ proteins known to date are present in MamJ-like, the N-terminal DXWX_2_LLEXSPWS and the C-terminal VPVEX_4_GXFX_2_AXSA motifs. With the online SCANPROSITE data mining tool (http://expasy.org/tools/scanprosite/), we searched UniProtKB/Swiss-Prot and UniProtKB/TrEMBL databases for these patterns and retrieved only MamJ sequences with an additional hit using solely the C-terminus pattern with the *amb1003* gene product (TrEMBL Q2W8L8) located in the MAI of *M. magneticum* AMB-1. Interestingly this predicted protein is situated between previously recognized MamE (TrEMBL Q2W8L9) and MamO (TrEMBL Q2W8L7) homologues within the MAI [13]. The predicted sequence for MamJ-like is somewhat shorter (375 residues for MamJ-like vs. 506 for MamJ) and lacks the characteristic acidic repeats of MamJ proteins [23]. Interestingly, when these repeats (as much as a 158-residue central stretch) are deleted from MamJ, mutant forms are still able to complement a *M. gryphiswaldense* MSR-1 Δ*mamJ* strain, restoring magnetosome alignment with the MamK cytoskeleton [22]. The length of these shortened protein sequences is similar to the length of MamJ-like.

**Table 3:** A new set of magnetotaxis-related genes outside the *M. magneticum* AMB-1 magnetosome island.

CDS (a)	Start (b)	Stop (c)	MAI (d)	% id. (e)	% sim. (f)	Gene (g)
*NEW_GENE*	425806	424787	MamK	54.5	71.5	*mamK-like*
*amb0400*	431795	432820	MamD	36.4	51.3	*mamD-like*
*amb0407*	438420*	438208	MamL	75.6	80.7	*mamL-like*
*MAGMM0452*	440560	439811	MamJ	15.9	29.2	*mamJ-like*
*amb0410*	443319**	440944	MamE	51.9	62.3	*mamE-like*
*amb0412*	445749	446084	MamF	59.4	73.0	*mamF-like*
*MAGMM0458*	446504	447079	MamQ	37.5	50.2	*mamQ-like*

a) Gene names used in the published genome of AMB-1 [8]. A new ORF we identified is temporarily referred to as *NEW_GENE*. b–c) Position of putative initiation codon on the bacterial chromosome. *, annotated initiation codon changed adding 18 residues to the gene product. **, annotated initiation codon changed adding 206 residues to the gene product. The entire MamE-like sequence is given in Figure S1. c) Position of stop codon on the bacterial chromosome. d) Homologous proteins encoded within the magnetosome island. e–f) Percentage of identity and similarity (BLOSSUM62 matrix) between putative proteins encoded by genes listed in a) and Mam proteins listed in d). g) Gene names used hereafter.

**Figure 1:**
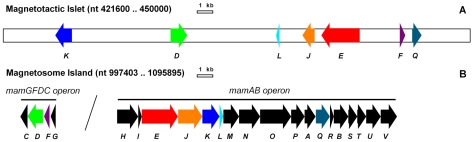
A magnetotaxis islet. Genetic organization of the magnetotaxis islet (MIS) compared to the magnetosome island (MAI) in *M. magneticum* AMB-1. Each *mam* gene in the MAI and its respective homologue in the MIS are shown in the same colour. Scale bars: 1 kb. A) The region including *mam-like* genes is situated between nucleotides (nt) 421600 and 450000 on the bacterial chromosome. For clarity ORFs unrelated to *mam* genes are not shown. B) Only the *mamGFDC* and *mamAB* operons from the MAI (nt 997403 to 1095895) are shown.

Among the seven ORFs, five are homologous to genes belonging to the *mamAB* operon (*mamE*, *mamJ*, *mamK*, *mamL* and *mamQ*) and the two remaining are similar to genes from the *mamGFDC* operon (*mamF* and *mamD*), both operons being located within the MAI. MamK and MamJ are involved in magnetosome alignment, MamE is a predicted serine protease (similar to DegP in *E. coli* and potentially involved in Fe^2+^-induced oxidative stress remediation [38]), and the molecular functions of MamL and MamQ are unknown. The hydrophobic MamD and MamF are found exclusively in the magnetosome membrane; they are involved in regulating magnetite crystal size though the underlying molecular mechanisms remain unknown [15]. The genetic organization of the *mam-like* genes differs from their MAI counterparts since the seven *mam-like* genes are scattered over a region of about 22 kb (Fig. 1).

The average GC content of the bacterial chromosome is 65.9% but this value drops to 52% when averaged over the seven *mam-like* genes alone (63.9% for the equivalent *mam* genes in the MAI). For instance the GC content is 67.6% for *mamE*, 68.5% for *amb1002* (*mamE* homologue within the MAI) but only 56.7% for *mamE-like*. Besides a lower GC content, numerous predicted transposase genes are interspersed with the *mam-like* genes (at least 6 annotated transposase sequences were found between *mamD-like* and *mamL-like*). Some of them are only fragments but genes encoding full-size proteins were identified by BLAST analysis, such as *amb0411* between *mamE-like* and *mamF-like*, *amb0408* between *mamJ-like* and *mamL-like*, and *MAGMM0433* between *mamK-like* and *mamD-like*. A set of genes originating from the bacteriophages D3 and MP29 from *Pseudomonas aeruginosa* can also be found in regions adjacent to the gene cluster; for instance, Amb0396 shares 44.9% identity with ORF22 from MP29 (TrEMBL B7SDT3) and Amb0447 49.2% identity with ORF7 from MP29 (TrEMBL B7SDR9). We identified a new ORF (from nt 448228 to nt 448884 on the chromosome) located at the end of the *mam-like* gene cluster (downstream of *mamQ-like*) encoding a putative ParA protein involved in plasmid partitioning. Taken together these genetic traits suggest that the 22-kb *mam-like* gene cluster forms a genomic islet, a term for small genomic islands [39], acquired by horizontal gene transfer or duplication of the MAI. We chose to name this locus the magnetotaxis islet (MIS). Similar islets were identified neither in the very closely related bacterium *M. magnetotacticum* MS-1, nor in *M. gryphiswaldense* MSR-1 nor in *Magnetococcus sp.* MC-1.

### Genes Belonging to the Magnetotaxis Islet Are Transcribed in *M. magneticum* AMB-1 Strains

**Figure 2:**
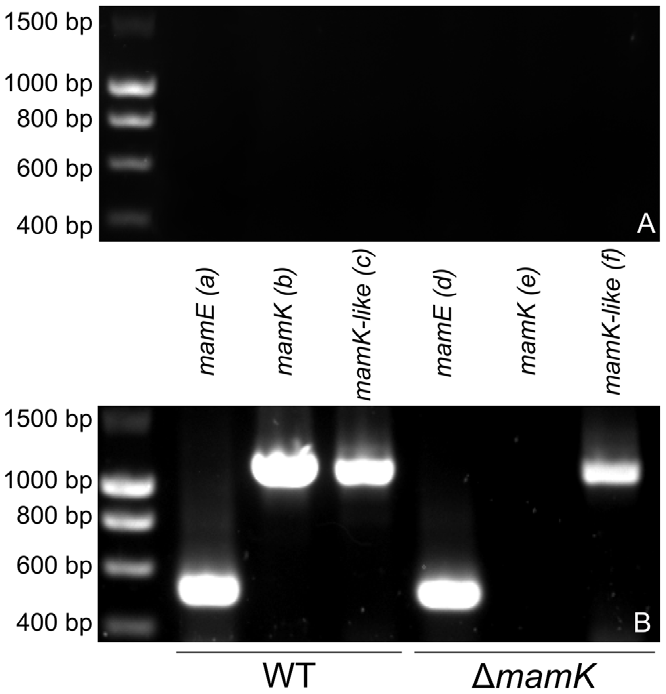
*mamK-like* transcription in *M. magneticum* AMB-1 WT and Δ*mamK* mutant strains. A) Negative controls (no RT). B) RT-PCR amplification from WT (a–c) and Δm*amK* (d–f) RNAs of *mamK* (b, e) and *mamK-like* (c, f). *mamE* was amplified (a, d) as an internal control. Sizes of DNA markers are given on the left on both panels.

This unexpected discovery of *mam* homologues outside the MAI prompted us to assess whether they are transcribed in AMB-1 cells. To date, the *mamK* gene product is the best characterized magnetotaxis protein and a deletion strain is available [27]. For these reasons we focused on *mamK-like* to test the expression of *mam-like* genes from outside the MAI in WT and Δ*mamK* strains by RT-PCR with primers designed to avoid cross-hybridization between *mamK* and *mamK-like* (see Materials and Methods). We used *mamE* as a positive control representative of the presence and expression of genes from the MAI. As shown in Fig. 2, *mamK-like* is transcribed in both strains, whereas *mamK* mRNA can only be detected in WT cells. We also found that *mamE-like* in the magnetotaxis islet and *mamE* from the MAI are transcribed in WT AMB-1 (data not shown). These results establish that *mamE-like* and *mamK-like* are not cryptic genes in a silent region of the bacterial chromosome but are expressed in AMB-1 cells in standard culture conditions. Do these genes have the same molecular properties as their MAI homologues?

### MamK-Like Is a Member of the Actin-Like Family

**Figure 3:**
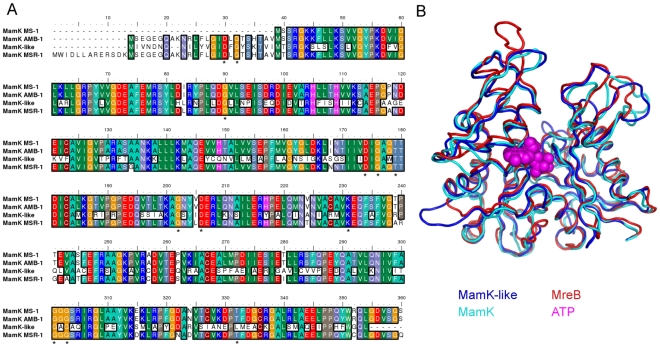
MamK-like belongs to the actin-like protein family. A) MamK homologue protein sequence alignment (ClustalW) for the *Magnetospirillum* genus. Accession numbers are Q2W8Q6 (AMB-1) and Q6NE59 (MSR-1) in TrEMBL, and ZP_00054405 (MS-1) in NCBI Refseq. *, conserved residues involved in ATP-binding. B) 3D-structural model of MamK-like (blue) and MamK (cyan) generated by 3D-JIGSAW using the structure of MreB (red) from *Thermotoga maritime* (PDB entry: 1JCF) as a template. The ATP molecule is shown in violet.

A protein sequence alignment of MamK proteins from *Magnetospirilla* including MamK-like is shown in Fig. 3*A*. The primary sequence of MamK-like is very similar to that of MamK (54.5% identical, see Table 3 and Fig. 3*A*). In addition, key residues putatively involved in the nucleotide-binding site are conserved (labelled residues in Fig. 3*A*, see [40]). Using a fully automated protein structure homology-modelling program, we determined putative models for MamK-like and MamK proteins (Fig. 3*B*, blue and cyan ribbons respectively). In both cases, MreB from *Thermotoga maritima* [40] is the closest structural template in the Protein Data Bank (PDB entry: 1JCF) automatically identified using sequence homology (Fig. 3*B*, red ribbon). The putative models contain the main conserved feature of all actin-like proteins, two domains in a characteristic fold (Fig. 3*B*) forming the nucleotide-binding site in the interdomain cleft (Fig. 3*B*, nucleotide in violet). Major differences between the three proteins are sequence insertions in loops which could be responsible for functional variations. Although experimental structural data are required for further analysis, these models show that both proteins are likely members of the prokaryotic actin-like family.

**Figure 4:**
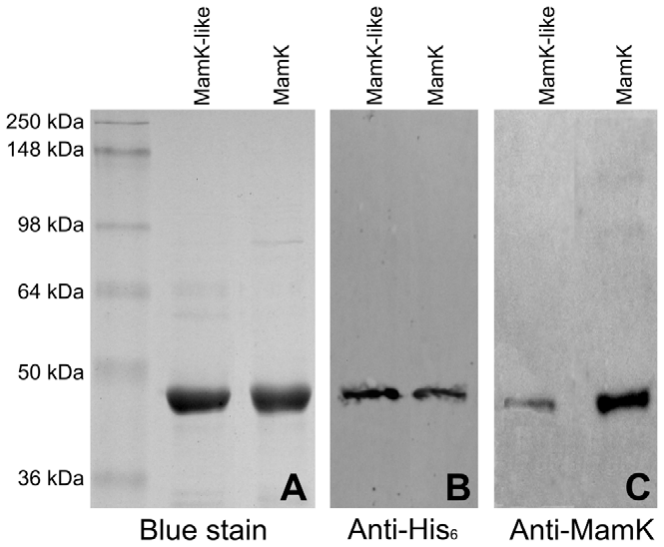
SDS-PAGE and Western blot detection of purified recombinant MamK-like and MamK. A) SDS-PAGE gel (10% acrylamide) with 2 µg of protein per lane stained with Coomassie blue. B) Western blot with 30 ng of protein per lane probed with anti-histidine tag antibody. C) Western blot with 30 ng of protein per lane probed with anti-MamK antibody. Molecular weights (MW) of protein standards are given on the left and apply to all panels. Expected MWs of MamK-like and MamK are 41 and 42 kDa respectively.

**Figure 5:**
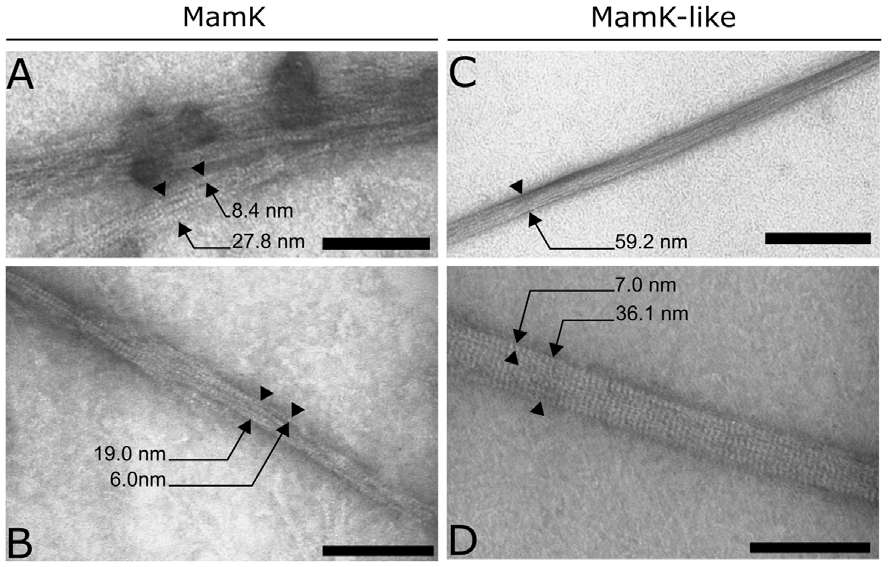
In vitro polymerization of MamK and MamK-like visualized by TEM. Sizes of structures are indicated with arrows. Structures narrower than 10 nm are termed “filaments” and larger structures are termed “bundles”. A–B) MamK polymers. C–D) MamK-like polymers. Scale bars: 100 nm in A, B and D; 300 nm in C.

Recombinant histidine-tagged MamK and MamK-like proteins were produced in *E. coli* and purified by metal affinity chromatography (Fig. 4*A*). Both purified proteins were detected by Western blot with an antibody raised against MamK (Fig. 4*C*), though detection of MamK-like was less sensitive. Size-exclusion experiments (data not shown) using different buffers revealed that addition of ATP is required to obtain stable monomers and to reduce polymerization and/or precipitation of both proteins during purification. To investigate this and test the ability of MamK and MamK-like to spontaneously form filaments, purified proteins were incubated in different buffers and analyzed after different time lapses by negative stain TEM. As previously shown for MamK [24], MamK-like is able to spontaneously polymerize into long straight filaments in the absence of ATP, a process triggered by the addition of KCl and MgCl_2_ (Fig. 5). MamK and MamK-like form large structures, we termed “bundles”, approx. 60 nm in width and reaching more than 1 µm in length. Smaller assemblies ranging from 20 to 35 nm in width can also be observed (Fig. 5). Small bundles are made up of individual filaments whose size (diameters from 6 to 8 nm) and striated appearance are consistent with them being elemental helical filaments described previously [24]. Small MamK and MamK-like bundles are organized differently. MamK bundles are composed of twisted filaments, whereas MamK-like filaments are assembled in a more regular, linear bundle. On a larger scale, well-developed bundles of MamK-like are also generally straighter and more regularly arranged than MamK bundles (Fig. 5*A,C*). Another major difference between the two proteins is in the in vitro polymerization kinetics since MamK-like bundles are only observed after 30 minutes of incubation whereas MamK polymerizes in less than 15 min.

**Figure 6:**
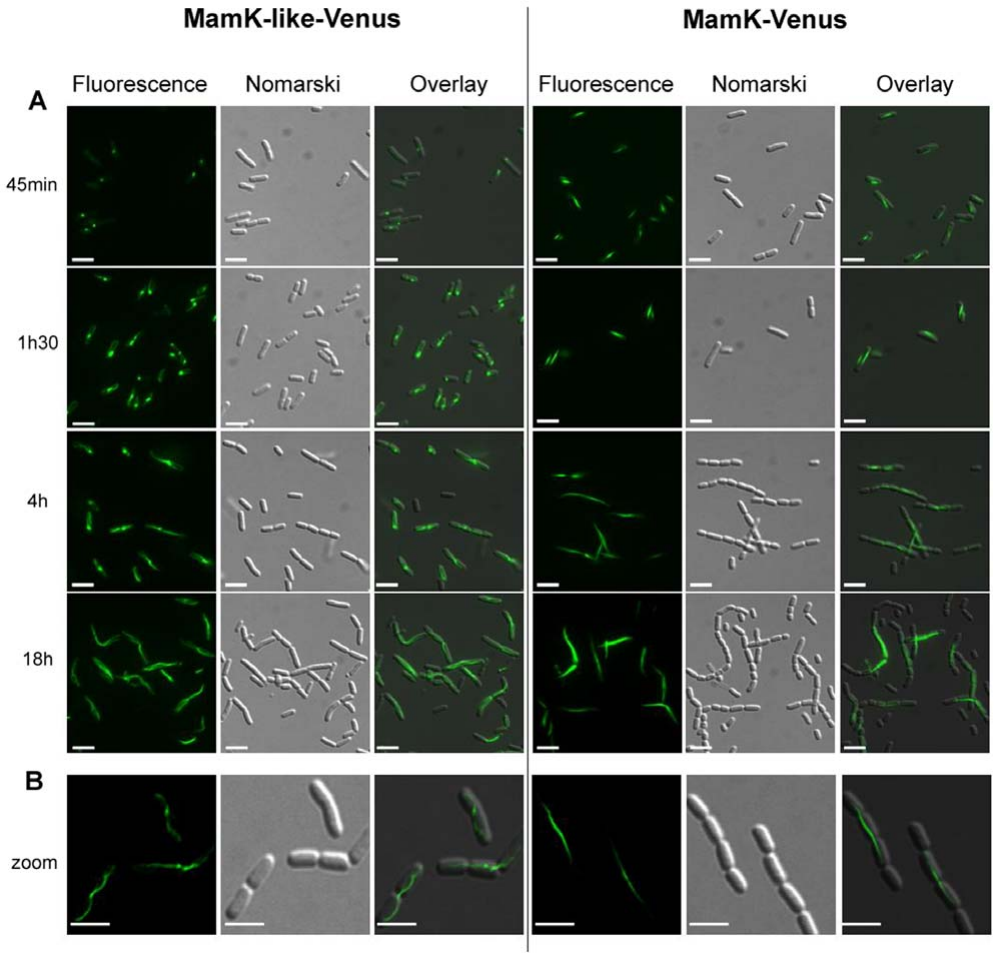
In vivo polymerization kinetics of MamK-like-Venus and MamK-Venus filaments in *E. coli* measured by fluorescence imaging. A) Polymerization kinetics. Protein production was induced by adding 0.1% arabinose during the exponential growth phase. Left panel, fluorescence emission at 528 nm (515 nm excitation); middle panel, Nomarski contrast; right panel, left and middle images overlaid. Scale bars: 5 µm. Time after induction is given on the left. B) Comparison of filament morphology of MamK-like-Venus and MamK-Venus after 3 h of induction with 0.2% arabinose. Left panel, fluorescence emission at 528 nm (515 nm excitation) after deconvolution; middle panel, Nomarski contrast; right panel, left and middle images overlaid. Scale bars: 3 µm.

In vivo polymerization kinetics of MamK and MamK-like in the form of GFP-variant fusion proteins were followed in *E. coli* as already described for MamK-GFP [20]. When expressed in *E. coli* TG1 as fusions with the fluorescent protein Venus, both MamK and MamK-like polymerize into long filaments in the cytoplasm (Fig. 6*A*, 18 h after induction), corroborating the purified protein data. As the reaction progresses chains of bacteria start to form, a sign that cell division is hindered. In both MamK- and MamK-like-Venus fusions, some filaments extend through several cell units (4 h after induction). However MamK-like-Venus polymerization differs significantly from that previously reported for MamK-Venus. MamK-Venus filaments nucleate at multiple sites and assemble into mosaic filaments, to form a single straight bundle which considerably increases in length and thickness during induction. One bundle per cell is visible 18 h after induction (Fig. 6*B*, right panel). By contrast, MamK-like-Venus first appears as a focus which is mostly located at one pole or septum of the cell (Fig. 6*A*, left panel, 45 min after induction). Then several thin twisted filaments emerge from this focus (Fig. 6*A*, left panel, 1 h 30 min and 4 h after induction). When polymerization ceases, the initial locus disappears (Fig. 6*A*, left panel, 18 h after induction) leaving very long, thin and twisted filaments (Fig. 6*B*, left panel). Unlike MreB [41], treatment of cells with S-(3,4-dichlorobenzyl) isothiourea (A22), an inhibitor of ATP binding, does not trigger depolymerization of MamK-like or MamK filaments (data not shown).

The *in-vitro* and *in-vivo* experiments described above demonstrate that MamK-like is an actin-like protein, able to polymerize into long straight filaments. As known for MamK, MamK-like polymerization is distinct from that of other bacterial actin-like proteins. Compared to MamK though, MamK-like filaments are thinner and polymerization is slower.

### Expression of MamK-Like in *M. magneticum* AMB-1 Cells

**Figure 7:**
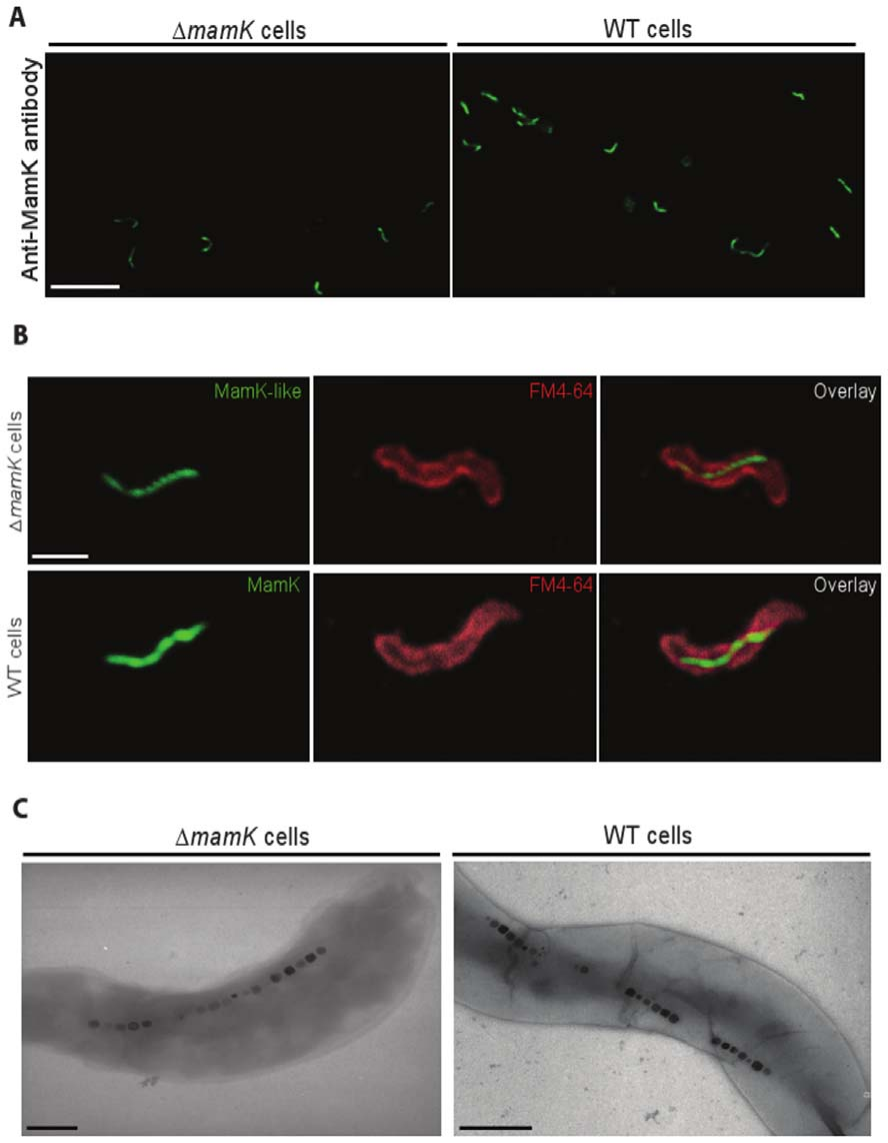
Detection of MamK and MamK-like filaments in fixed cells of *M. magneticum* AMB-1 WT and Δ*mamK* strains. The primary antibody was the anti-MamK antibody and the secondary antibody was coupled to FITC. Confocal microscope data acquisition parameters: 3D stacks of 2048 x 2048 pixel images, 0.16 µm steps, 4x frames average, 2x line accumulation. A) FITC fluorescence emission at *λ* = 518 nm (excitation at *λ* = 488 nm), 2.4x microscope zoom. Left panel, Δ*mamK* mutant cells; right panel, WT cells. Scale bar: 10 µm. B) Cell membranes were stained with FM® 4-64 FX. Upper panels, Δ*mamK* mutant cells; lower panels, WT cells. Left column, FITC fluorescence emission at *λ* = 518 nm (excitation at *λ* = 488 nm); middle column, FM® 4-64 FX fluorescence emission at *λ* = 744 nm (excitation at *λ* = 633 nm); right column, left and middle column images overlaid; 4.74x microscope zoom for all panels. Scale bar: 1 µm. C) Visualization of magnetosome alignment with TEM. Left panel, Δ*mamK* cells (unstained); right panel, WT cells (1% uranyl acetate stained). Scale bar: 300 nm.

Knowing that *mamK-like* is transcribed in AMB-1 WT and Δ*mamK* strains, and that the recombinant protein polymerizes to form bundles of filaments, we further investigated the expression of MamK and MamK-like proteins in AMB-1 cells using immunofluorescence. Notwithstanding the high degree of similarity between the two proteins, the existence of the MamK-like isoform raises the question as to whether its localization and organization within AMB-1 WT cells is the same as for MamK? Using the *mamK* deletion strain allowed us to investigate MamK-like expression independent of MamK. The absence of the *mamK* gene from the mutant was confirmed by PCR analysis (data not shown). We also verified that the anti-MamK antibody can indeed detect MamK and MamK-like filaments in vivo in *E. coli* cells expressing the different *mamK* constructs (see Figure S2). We then observed MamK-like filaments in AMB-1 WT (Fig. 7*A*, right panel). Surprisingly, an actin-like filament was detected with the same antibody in *M. magneticum* AMB-1 Δ*mamK* (left panel). Furthermore, filaments observed in the mutant are organized as in WT cells, spanning the cytoplasm from pole to pole (Fig. 7*B*). They are thinner than MamK filaments, consistent with the properties we observed for recombinant protein polymerization and the expression of variant GFP-fusion proteins (Fig. 5 and 6). These findings uphold the hypothesis that the filaments observed in the mutant are polymers of MamK-like. Interestingly, the presence of an actin-like filament in the AMB-1 Δ*mamK* mutant corroborates the observation that magnetosomes are in a chain-like configuration (Fig. 7*C*) in this strain despite the lack of MamK [27], [28].

## Discussion

### *mam* Gene Redundancy Outside the MAI: Remnants from the Origins of Magnetotaxis?

Finding MTB-related genes outside the MAI brings a new perspective to the genetic study of how magnetotaxis has been acquired by microorganisms. Redundancy between magnetotaxis-related genes has been reported, although little is known about its physiological significance. A series of gene duplications have already been identified within the MAI of MTBs. For example, *mamE*-, *mamO*-, *mamQ*-, *mamR*-, *mamB*- and *mamF-like* genes in *M. magneticum* AMB-1; a *mamK*- and a *mamH-like* gene in the MAI of marine magnetotactic vibrio strain MV-1; and similar duplications in two recently sequenced MAI from environmental samples (for a comprehensive view see [13]). Our analysis also suggests there is a previously unreported homologue of *mamJ* located between *mamE-* and *mamO-like* genes in AMB-1 MAI (*amb1003*).

Several genetic features point to the theory that this magnetotaxis islet was acquired by horizontal gene transfer, rather than by simple genetic rearrangements with the MAI. Besides having a lower GC content, there are numerous transposable elements and bacteriophage-related genes within or in the vicinity of the MIS. As explained by Schübbe et al. [9] the integration of prophage genes into MTB genomes may be an additional cause of genetic instability and lateral gene transfer. Understanding the exact mechanism by which this MIS has been acquired by *M. magneticum* AMB-1 is beyond the scope of this paper, but the genetic traits are suggestive of the DNA region being very mobile at least in the evolution of AMB-1.

**Figure 8:**
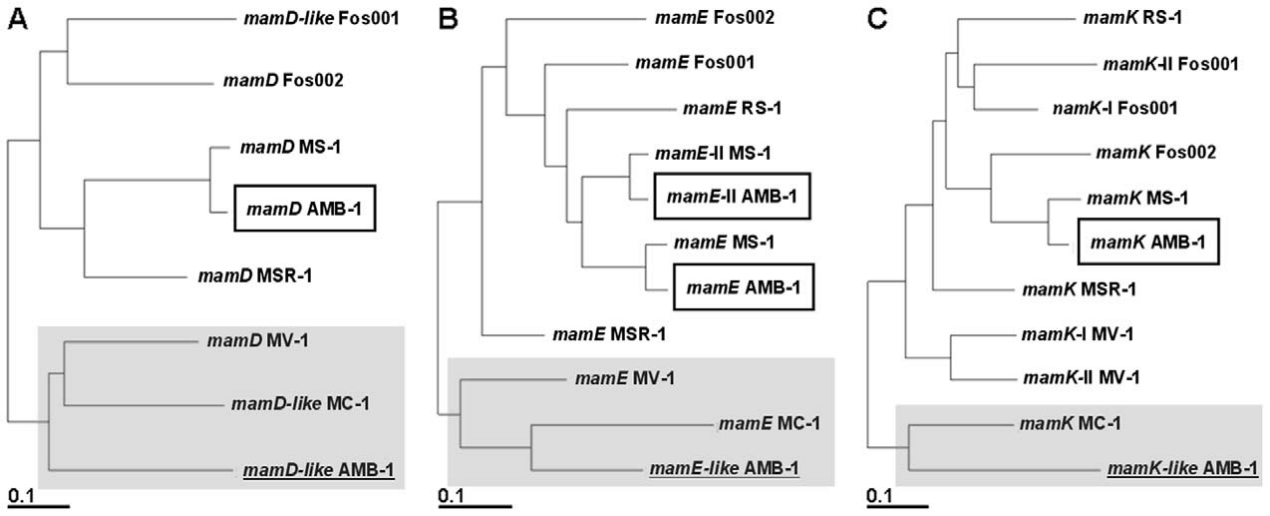
General codon usage analysis. RSCU-based trees generated with the Fitch-Margoliash distance-based optimization method. The best trees were computed by the algorithm from 100 randomized input order distance matrixes. A) *mamD* gene family. B) *mamE* gene family. C) *mamK* gene families. MamE-II (for MS-1 and AMB-1) are MamE homologues encoded in the MAI. Sub-trees comprising *mam-like* genes from the AMB-1 magnetotaxis islet are shaded grey. Boxed gene names are the *mam* genes belonging to the AMB-1 magnetosome island. Accession numbers for the encoded proteins are listed in Table S1.

Tracing back the origin of this MIS is no simple matter. Most of the genes encoded are MTB-specific and as a consequence there is a very limited number of homologous genes and proteins to work with (11 sequences for *mamK* but only 4 sequences for *mamJ* which to date has only been found in *Magnetospirilla*). The significant difference in GC content prompted us to examine the codon usage pattern in the MIS and compare it with those of other known *mam* or *mam-like* genes. We generated phylogenetic trees allowing us to group these genes based on codon usage, at least for the 5 *mam-like* genes universally shared by MTBs, i.e. *mamE-, mamK-, mamQ-, mamD- and mamF-like* (only *Desulfovibrio magneticus* RS-1 is devoid of *MamD*). Although statistical bias may be introduced using only short sequences (*mamF-like* for instance with only 112 codons) there was good accordance between all trees thus constructed. Fig. 8 shows the codon usage-based phylogenetic trees built for *mamD-*, *mamE-* and *mamK-like* (for the 4 remaining *mam-like* genes, data not shown). Without exception *mam-like* genes group together with *Magnetococcus sp.* MC-1 homologues, as well as in most cases with those from marine magnetotactic vibrio strain MV-1 (shaded boxes in Fig. 8). MAI genes from *Magnetospirilla* are grouped together. This result clearly establishes the common origin of the genetic material from which MAIs from MC-1 and MV-1 and the MIS from AMB-1 evolved.

**Figure 9:**
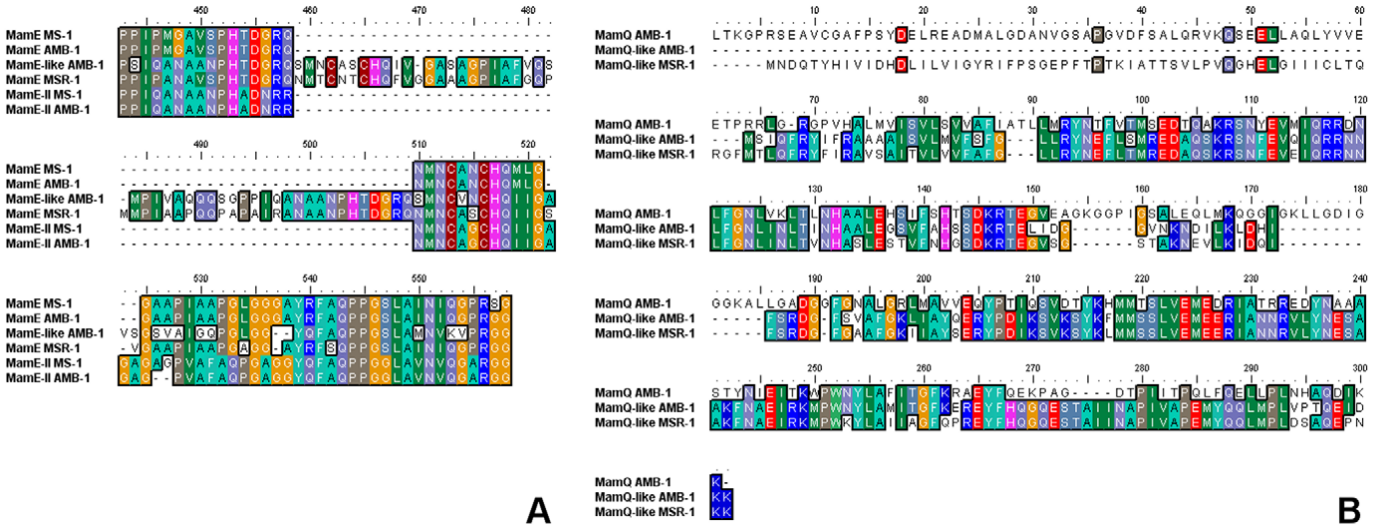
Kinship of MIS *mam-like* genes with MAI *mam* genes of *M. gryphiswaldense* MSR-1. A) Partial sequence alignment of MamE proteins from the *Magnetospirillum* genus. B) Protein sequence alignment between MamQ-like encoded in the MIS of AMB-1, the newly identified MamQ-like from MSR-1 (MGR_0326) and MamQ encoded in the MAI of AMB-1.

In terms of reflecting protein evolution, phylogenetic trees based on protein sequence alignments are much more difficult to build and interpret than codon usage-based trees. Nevertheless both methods concur that sequences of MIS Mam-like proteins tend to cluster with protein sequences encoded in the MAI of *Magnetospirilla*, and are very distant from MC-1 Mam protein sequences (data not shown). Only inspection of protein alignments “by eye” yielded useful information from which to infer phylogenetic relationships in the *Magnetospirillum* sub-tree, as illustrated by MamE-like. The predicted MamE-like protein is more similar to MAI MamE of MSR-1 (56.3% identity) than the equivalent in AMB-1 (51.2% identity). Furthermore, there is a stretch of 50 residues in MamE-like (from Asn434 to Gln484) which is absent in MamE of AMB-1 and MS-1, but present in MamE of MSR-1 with 68.6% identity (Fig. 9*A*). MamQ-like is also very similar to MGR_0326 (63.5% identity, Fig. 8*B*), an MSR-1 protein whose similarity with MamQ has not been reported before; the identity between MamQ-like and MamQ in AMB-1 is only 37.5% (Fig. 9*B*). We searched for a genomic islet in the vicinity of *MGR_0326* but could not find any additional Mam-like proteins in the *M. gryphiswaldense* MSR-1 genome. *MGR_0326* is close to the *mreBCD* gene cluster (*MGR_3222* to *MGR_3224*) involved in rod-shape determination. Naturally such a cluster exists in *M. magneticum* AMB-1 (*amb3513* to *amb3515*) but no nearby MamQ-like protein could be found. For MamK, examination of sequence alignments of proteins belonging to the *Magnetospirillum* genus reveals that MamK-like is more closely related to the MSR-1 protein (see positions 40, 83, 118 and 257 in MSR-1, see Fig. 3*A*). When taken together these analyses suggest that Mam-like proteins encoded in the MIS evolved in a similar fashion to those encoded within the MAI of MSR-1, rather than of AMB-1.

These observations substantiate the hypothesis that the MIS in AMB-1 was acquired independently by horizontal gene transfer. It is puzzling that MS-1 seems to be devoid of this genetic islet given its phylogenic proximity to AMB-1. Either this locus has been lost in MS-1 or was acquired by AMB-1 after they diverged. Jogler et al. [13] proposed separate horizontal gene transfer events could account for magnetotaxis in organisms like MC-1 and AMB-1 that live in distinct environments (marine vs. fresh water). The common genetic background of the MAI in MC-1 and the MIS in AMB-1 that we postulate in this study would imply that their ancestors must have come into contact with the same “magnetotaxis gene provider”, and therefore must have evolved in the same habitat before specializing in marine and fresh waters. MC-1 would have then lost genes such as *mamL* and *mamJ* only found in *Magnetospirillum* MAI and in the MIS. The latter could be a remnant of an ancestral magnetotaxis-specific set of genes, most probably reshuffled many times, accounting for the very few genes remaining in this islet when compared to the 17 magnetotaxis-specific genes identified elsewhere. The actual minimal number of genes required for magnetosome synthesis has yet to be determined, as MTB-specific genes like those of the *mamGCDF* operon can be deleted without suppressing magnetite biomineralization [15]. The existence of magnetotaxis-specific genes outside the MAI does raise the question of their role in AMB-1 physiology. We demonstrated that two of these genes, *mamK-* and *mamE-like*, are transcribed in standard culture conditions. This hints at the possibility that the entire MIS is expressed in parallel with MAI expression. Supposing the MIS *mam-like* gene products are functional, interactions with proteins encoded in the MAI are likely to occur. Of the seven *mam-like* genes we identified in the MIS, the *mamK-like* gene was the best candidate for molecular studies.

### MamK-Like Is a Member of the Actin-Like Protein Family

Sequence analysis and molecular modelling show that MamK-like, along with MamK, belongs to the prokaryotic actin-like protein family. In vitro polymerization assays, as well as in vivo fluorescence imaging show that MamK-like polymerizes into long filaments, as previously shown for MamK. Prokaryotic actin homologues are involved in a variety of essential cellular processes in bacteria, from rod-shape determination (MreB) to plasmid segregation (ParM) and pseudo-organelle alignment (MamK). MreB was the first discovered and is the best characterized member of this family (for reviews see [21], [22]). The structure of the monomer has been solved by X-ray diffraction, the ultrastructural organization of the filaments has been characterized in vitro and in vivo, and the dynamics of polymerization have been investigated biochemically and with electron microscopy. MreB was shown to have a 3D structure remarkably similar to that of actin and to undergo actin-like polymerization. Based on a fully automated protein structure homology-modelling program, our data suggest that MamK and MamK-like proteins adopt a fold as in MreB and actin. However, unlike MreB, MamK and MamK-like filaments do not require ATP for polymerization [24]. Treatment of MreB filaments with A22 triggers depolymerisation, whereas no effect was seen with MamK and MamK-like. MreB was shown to form predominantly filamentous bundles that spontaneously form ring-like structure, whereas both MamK and MamK-like polymerize in long linear structures. However bundles formed by each protein differ in how they are organized at the molecular scale: MamK bundles are twisted assemblies of filaments, MamK-like bundles appear as straight assemblies, although the basic structure of the filament looks conserved (striated, with similar diameter). In *E.coli*, MamK and MamK-like bundles differ regarding their spatial organisation and polymerization process: MamK-like bundles are more twisted and emerge from an unique focus, whereas MamK bundles are straighter and emerge from multiple foci. Nevertheless, MamK and MamK-like filament bundles, as observed by immunofluorescence, appear rather similar in *Magnetospirillum* cells. Further structural and functional studies of MamK proteins are required to understand how their specificities relate to the properties of the cytoskeletal filamentous structure associated with magnetosome chains.

We showed that the MamK-like protein is synthesized in a mutant lacking *mamK*. Based on mutant phenotypes, magnetosome alignment is thought to occur via the interaction of the MamK cytoskeleton with MamJ, a protein located in the magnetosome vesicle. However, the phenotypes of deletion strains are significantly different between *Magnetospirilla* species. Thus in *M. magneticum* AMB-1 Δ*mamK* strain, magnetosomes are still synthesized but are in a dispersed, chain-like configuration, whereas in the *M. gryphiswaldense* MSR-1 Δ*mamJ* mutant, magnetosomes are clustered and completely disorganized within the cell [25]. If a series of magnets were free to diffuse, one would expect precisely such an outcome due to basic magnetic attraction. The lack of the MamK filament to provide the scaffold for magnetosome alignment could perhaps elicit similar effects. Komeili and Schüler [27], [28] have described the Δ*mamK* phenotype in AMB-1 as being less clear-cut than expected and to our knowledge there is currently no Δ*mamK* mutant in *M. gryphiswaldense* MSR-1. The existence of a thin MamK-like filament in AMB-1 could maintain a chain-like organization of magnetosomes, thus accounting for the variable phenotype of the Δ*mamK* mutant. A double mutant strain Δ*mamK* and Δ*mamK*-like together with specific antibodies raised against each protein will allow us to prove any MamK-like involvement in magnetosome organization. There is a discrepancy between the phenotype described by Komeili et al. [19] and ours (present work) for the Δ*mamK* mutant. In our hands the fraction of misaligned magnetosomes chains in Δ*mamK* is well within the variability usually observed in the wild-type strain. It is possible that the previously published data represent extreme phenotypes of the Δ*mamK* mutant we didn't meet in our laboratory's conditions.

To summarize, the discovery of a magnetotaxis islet gives new insight into the origins of magnetotactic behaviour in bacteria. If like *mamK-like*, genes in this islet are active and encode functional *mam* homologues then new elements must be added to the model of how the magnetosome works.

## Supporting Information

Table S1 Accession numbers for proteins whose genes appear in Figure 8. * We redefined the initiation codon for this gene, adding 206 residues to the registered protein sequence. This revised sequence is shown in Figure S1. MamE-II for *M. magneticum* AMB-1 and *M. magnetotacticum* MS-1 refer to MamE homologues proteins situated in the Magnetosome Island.
(0.06 MB DOC)

Figure S1 Protein sequence of *M. magneticum* AMB-1 *mamE-like* gene product situated in the Magnetotactic Islet. We modified *amb0410* (TrEMBL Q2WAB1) initiation codon, yielding *mamE-like* gene. The corresponding protein is 206 residues longer.
(0.02 MB DOC)

Figure S2 Visualization of MamK-like and MamK filaments in *E. coli* by immunofluorescence imaging. *E. coli* cells express histidine-tagged constructs. Primary antibody is an anti-MamK antibody (rabbit); secondary antibody is an anti-rabbit IgG (mouse) coupled to TRITC. Scale bare is 3.5 µm.
(0.28 MB DOC)
