# Supplementary material for: A Second Actin-Like MamK Protein in Magnetospirillum magneticum AMB-1 Encoded Outside the Genomic Magnetosome Island
Source: PLoS One. 2010 Feb 10;5(2):e9151. doi: 10.1371/journal.pone.0009151 (PMC2818848; doi:10.1371/journal.pone.0009151)
Supplement: Table S1 — Accession numbers for proteins whose genes appear in Figure 8. * We redefined the initiation codon for this gene, adding 206 residues to the registered protein sequence. This revised sequence is shown in Figure S1. MamE-II for M. magneticum AMB-1 and M. magnetotacticum MS-1 refer to MamE homologues proteins situated in the Magnetosome Island. (0.06 MB DOC) [file pone.0009151.s001.doc]

| **Protein name** | **Organism** | **Database** | **Accession number** |
| --- | --- | --- | --- |
| MamD | AMB-1 | TrEMBL | Q2W8R9 |
| MamD | Fos002 | Genbank | CAX84209.1 |
| MamD | MS-1 | NCBI RefSeq | ZP_00053415.1 |
| MamD | MSR-1 | Genbank | AAL10003.1 |
| MamD-like | AMB-1 | TrEMBL | Q2WAC1 |
| MamD-like | Fos001 | Genbank | CAX83780.1 |
| MamD-like | MC-1 | TrEMBL | A0L9U5 |
| MamD-like | MV-1 | Genbank | CAV30792.1 |
| MamE | AMB-1 | Genbank | BAE49767.1 |
| MamE | Fos001 | Genbank | CAX83785.1 |
| MamE | Fos002 | Genbank | CAX84228.1 |
| MamE | MC-1 | NCBI RefSeq | YP_866174.1 |
| MamE | MS-1 | NCBI RefSeq | ZP_00054403.1 |
| MamE | MSR-1 | TrEMBL | Q6NE61 |
| MamE | MV-1 | Genbank | CAV30818.1 |
| MamE | RS-1 | NCBI RefSeq | YP_002955485.1 |
| MamE-II | AMB-1 | Genbank | BAE49806.1 |
| MamE-II | MS-1 | NCBI RefSeq | ZP_00053527.1 |
| MamE-like* | AMB-1 | TrEMBL | Q2WAB1 |
| MamK | AMB-1 | TrEMBL | Q2W8Q6 |
| MamK | Fos002 | Genbank | CAX84225.1 |
| MamK | MC-1 | TrEMBL | A0L9W7 |
| MamK | MS-1 | NCBI RefSeq | ZP_00054405.2 |
| MamK | MSR-1 | TrEMBL | Q6NE59 |
| MamK | RS-1 | Genbank | BAH77585.1 |
| MamK-I | Fos001 | Genbank | CAX83788.1 |
| MamK-I | MV-1 | Genbank | CAV30816.1 |
| MamK-II | Fos001 | Genbank | CAX83791.1 |
| MamK-II | MV-1 | Genbank | CAV30798.1 |
| MamK-like | AMB-1 | Genbank | GQ457518 |
