# Supplementary material for: A Second Actin-Like MamK Protein in Magnetospirillum magneticum AMB-1 Encoded Outside the Genomic Magnetosome Island
Source: PLoS One. 2010 Feb 10;5(2):e9151. doi: 10.1371/journal.pone.0009151 (PMC2818848; doi:10.1371/journal.pone.0009151)
Supplement: Figure S1 — Protein sequence of M. magneticum AMB-1 mamE-like gene product situated in the Magnetotactic Islet. We modified amb0410 (TrEMBL Q2WAB1) initiation codon, yielding mamE-like gene. The corresponding protein is 206 residues longer. (0.02 MB DOC) [file pone.0009151.s002.doc]

MFNRSADDEGHSNATRSKELKRYLMLMGVIALIVLFGAFIYRQPSGGLRL

ESILEQLGHGSSVNLPTQPGAVARLQPNTGGVPRALASSGGGFSNVAALL

RNGIVSVKASSPSRQPALGNFALDHQSTTPRFANPSASFVDSVGTGVIVR

DEGFIITNFHVVRGAQSVFVTVPDDTGSTRYSAEIVKLDEALDLALLKIT

PKAQLSVAVLGNSDAVLVADEVIAIGTPFGLDMSVSRGIISAKNKSMVIE

GVTHSKLLQTDAAINQGNSGGPLVIANGTVIGINTAIYTPNGAFSGIGFA

VPSNQARQFAQEVVGWLPTTTAEGPSMGLVALQGPSRIGVGSAGPPIMAR

MSSPHTDGRQNLDCANCHEILPSNPGVAAAGSLMPVANPRLPPPIRAIAT

PPHTDGRQNMACNTCHQIIGAPAGRVAFTQPMIPIVAGQPAGPSIQANAA

NPHTDGRQSMNCASCHQIVGASAGPIAFVQSMPIVAQQQSGPPIQANAAN

PHTDGRQSMNCVNCHQIIGAVSGSVAIGQPGLGGYQFAQPPGSLAMNVKV

PRGGQAARETYPNHNLLGAFMPPMSQLMGGQINVPAGRGVFITGVSPNTP

AAAAGLQAGDMLLKVDGRPVNSAREVIAIISEMPNGRSVRLGILREGSVS

NLTLLVGPTAAAPPAPKQNAGAGNNNVVQPAAPAKPAKPPKIPTEFTWMG

MEIDGFMEQPMMSGSPDEPPIKTTGAQVAEMATGSRAEAAGVMPNDLILE

VNNMPVTTPALLDAAIKAAKTAGPEVLLRVNRSGREVWVVL
