# Supplementary figures and images for: A Second Actin-Like MamK Protein in Magnetospirillum magneticum AMB-1 Encoded Outside the Genomic Magnetosome Island
Source: PLoS One. 2010 Feb 10;5(2):e9151. doi: 10.1371/journal.pone.0009151 (PMC2818848; doi:10.1371/journal.pone.0009151)

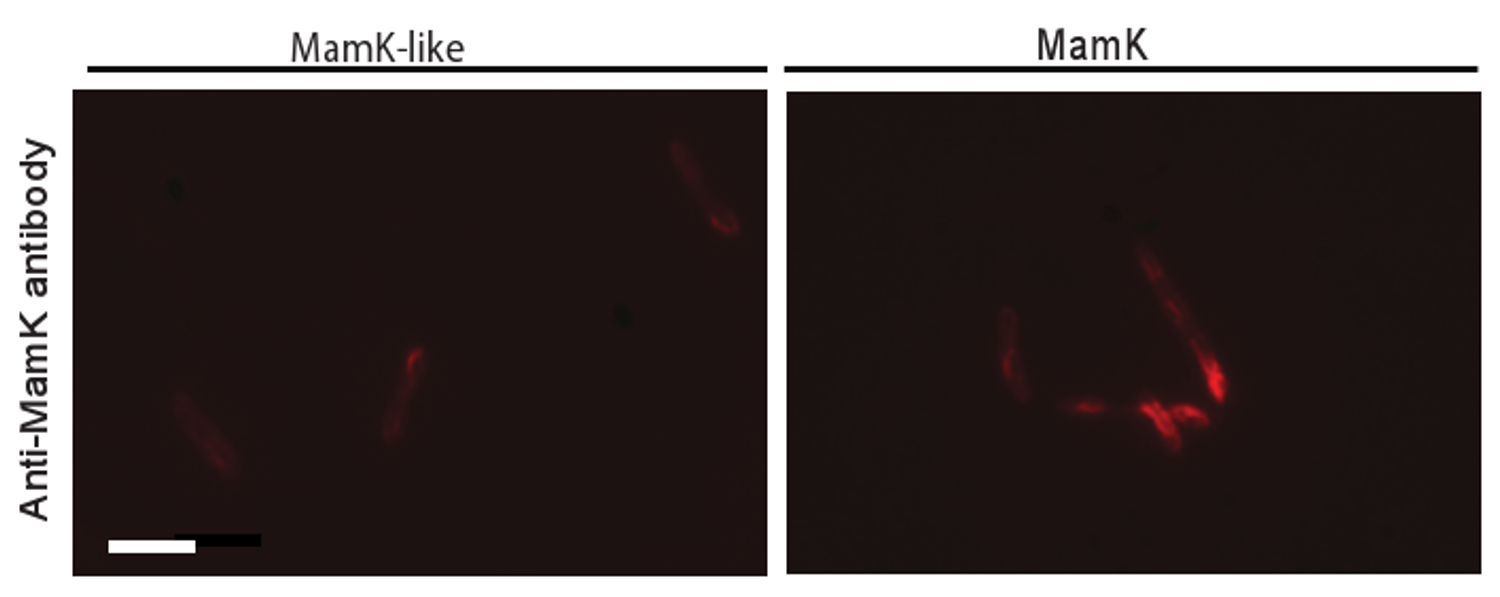

Supplement: Figure S2 — Visualization of MamK-like and MamK filaments in E. coli by immunofluorescence imaging. E. coli cells express histidine-tagged constructs. Primary antibody is an anti-MamK antibody (rabbit); secondary antibody is an anti-rabbit IgG (mouse) coupled to TRITC. Scale bare is 3.5 µm. (0.28 MB DOC) [file pone.0009151.s003.doc]
